# Infrequent Loss of Luminal Differentiation in Ductal Breast Cancer Metastasis

**DOI:** 10.1371/journal.pone.0078097

**Published:** 2013-10-21

**Authors:** Julia Calvo, Lourdes Sánchez-Cid, Montserrat Muñoz, Juan José Lozano, Timothy M. Thomson, Pedro L. Fernández

**Affiliations:** 1 Department of Pathology, Hospital Clínic, Barcelona, Spain; 2 Department of Cell Biology, Molecular Biology Institute of Barcelona (IBMB), National Research Council (CSIC), Barcelona, Spain; 3 Department of Medical Oncology, Hospital Clínic, Barcelona, Spain; 4 Department o de Anatomia Patológica, Farmacología y Microbiología, University of Barcelona, Barcelona, Spain; 5 Plataforma de Bioinformática, Centro de Investigación Biomédica en Red de Enfermedades Hepáticas y Digestivas (CIBER-EHD), Hospital Clinic, Barcelona, Spain; 6 Plataforma de Bioinformática, Centre d’ Investigacions Esther Koplowitz,; (CEK), Barcelona, Spain; 7 Centro de Investigación Biomédica en Red en Bioingeniería, Biomateriales y Nanomedicina (CIBER-BBN), Barcelona, Spain; 8 Institut d’Investigacions Biomèdiques August Pi i Sunyer, Barcelona, Spain; University of North Carolina School of Medicine, United States of America

## Abstract

Lymph node involvement is a major prognostic variable in breast cancer. Whether the molecular mechanisms that drive breast cancer cells to colonize lymph nodes are shared with their capacity to form distant metastases is yet to be established. In a transcriptomic survey aimed at identifying molecular factors associated with lymph node involvement of ductal breast cancer, we found that luminal differentiation, assessed by the expression of estrogen receptor (ER) and/or progesterone receptor (PR) and GATA3, was only infrequently lost in node-positive primary tumors and in matched lymph node metastases. The transcription factor GATA3 critically determines luminal lineage specification of mammary epithelium and is widely considered a tumor and metastasis suppressor in breast cancer. Strong expression of GATA3 and ER in a majority of primary node-positive ductal breast cancer was corroborated by quantitative RT-PCR and immunohistochemistry in the initial sample set, and by immunohistochemistry in an additional set from 167 patients diagnosed of node-negative and –positive primary infiltrating ductal breast cancer, including 102 samples from loco-regional lymph node metastases matched to their primary tumors, as well as 37 distant metastases. These observations suggest that loss of luminal differentiation is not a major factor driving the ability of breast cancer cells to colonize regional lymph nodes.

## Introduction

 Morphological and molecular subtypes of breast cancer have been associated with distinct stages of normal epithelial differentiation [1]. The most common morphological types of breast cancer are ductal and lobular infiltrating carcinomas [2]. Intrinsic molecular classifications based on transcriptomic analysis provide additional knowledge as to the biological basis of breast cancer heterogeneity and the corresponding putative cells of origin. Based on specific sets of markers, the four major molecular types of breast cancer are luminal A, luminal B, basal and HER2-enriched [3,4]. Molecular classifications of breast cancer can afford prognostic indicators independent of morphological assessment [5-7]. However, morphological diagnosis of breast cancer subtypes maintains a prevalent use in many clinical settings mostly because of considerations of cost effectiveness and prompt diagnosis and classification. Furthermore, when combined with selected molecular markers, including the immunohistochemical detection of hormone receptors, HER2 and the proliferation marker Ki67 and copy number quantification of the HER2 locus by FISH, it provides adequate information for the therapeutic management of the tumors, as well as reasonable prognostic value, probably not inferior to molecular classifications for the most common types of breast cancer [8-11]. 

In addition to molecular and morphological classifications, histological grade is a prognostic indicator [12,13], That breast cancer histological grades have a biological basis is supported by the strong molecular correlates associated with each discrete grade [14], suggesting that expert application of appropriate morphological criteria can extract biologically and clinically relevant information. In molecular terms, less differentiated luminal breast cancers, and thus higher histological grade tumors, are expected to express lower levels of luminal lineage differentiation markers, including estrogen receptor and GATA3.

A third major prognostic variable in breast cancer is lymph node involvement at the time of diagnosis and/or first surgery [12,15,16], usually appraised along with tumor size, although these two parameters may not be necessarily linked mechanistically [17]. Lymph node involvement and tumor size provide prognostic information independent of molecular or morphological classifications [18]. Despite the prognostic importance of lymph node involvement, comparatively little is known about the molecular mechanisms that endow breast cancer cells with the capacity to metastasize to regional lymph nodes [19], as opposed to molecules identified as involved in breast cancer distant organ colonization [20]. Also, whether lymph node metastasis and distant organ metastasis reflect common or differentiated biological properties of tumor cells is disputed [19,21], with evidences for and against lymph node involvement reflecting general metastatic potentials of breast cancer cells or representing a step that precedes distant organ dissemination in linear models of breast cancer evolution [22].

We report here that the expression of the breast luminal differentiation markers estrogen receptor and GATA3 in metastatic ductal breast cancers with a luminal phenotype is not generally lost or decreased upon regional or distant metastasis. Our observations suggest that loss of luminal differentiation is not a frequent process associated with the ability of luminal breast cancer cells to colonize regional lymph nodes or distant metastatic sites.

## Materials and Methods

### Ethics Statement

Patient selection and sample procurement complied with Spanish laws regarding data protection and written informed consent, which was obtained from all patients and stored at the Hospital Clinic Biobank, and were approved by the Hospital Clinic and IDIBAPS Ethics Committee and Review Board.

### Sample procurement and selection

For transcriptomic analysis, we selected untreated grade 2 or 3 infiltrating ductal carcinoma cases, 7 primary tumors without axillary lymph node involvement at presentation (primary, non-metastatic, PNM), 18 primary tumors with at least one affected axillary lymph node (primary, metastatic, PM), and their matching tumor-infiltrated lymph nodes (LNM). Samples were embedded in OCT compound (Sakura Finetec, Zoeterwoude, Nederland), snap-frozen in isopentane and stored at - 80 °C until use. Only samples with at least 70% tumoral epithelium versus stroma ratios were selected for the study. Three non-metastatic (normal) axillary lymph nodes(LN(-)) from three of the above tumors with metastases in other nodes were also included as controls.

 For immunohistochemical analysis, tissue microarrays (TMA) were built from paraffin-embedded samples, bearing 3-µm thick, 1-mm diameter duplicate or triplicate cores for each of 52 samples from node-negative primary tumors, 115 node-positive primary tumors, 111 lymph node metastases, 36 from distant metastases (16 lung, 5 pleura, 3 liver, 2 brain, peritoneum and skin, and one from adrenal gland, cerebellum, meninx, muscle, ovary and trachea), 7 ductal carcinoma in situ (DCIS) and 11 normal breast samples corresponding to normal epithelium from cases of primary tumors. All primary tumors were of ductal type and 11 were histological grade 1, 111 grade 2 and 45 grade 3. Primary tumors were classified according to the expression of luminal markers and HER2 (Table S1). Cases from the initial molecular analysis were also included in immunohistochemical analysis.

### RNA extraction and microarray analysis

Total RNA was extracted from 20-30 µm cryosections with the RNeasy Mini Kit (Qiagen, Hilden, Germany) followed by DNAse treatment, and quality and concentration were assessed with the 2100 Agilent Bioanalyzer (Agilent Technologies, Santa Clara, CA). Total RNA was retrotranscribed to cDNA, transcribed to cRNA, labeled with biotin and hybridized to Human Genome U133 A2.0 Arrays (Affymetrix, Santa Clara, CA). Microarray data were normalized using the robust multi-array (RMA) algorithm [23]. Next, those probes with a maximum expression value lower than 5 were eliminated. Significance Analysis of Microarrays (SAM-R) [24] was applied to identify differentially expressed genes, selecting those genes with a False Discovery Rate (FDR) < 10%. Gene co-regulation was determined as the strength of the association between two gene expression profiles, for which Pearson correlation indexes (r) were computed between a preselected probelist versus the rest of filtered probes, using the cor.test function from the R-package. Microarray data are available at GEO accession GSE44408.

### Real-time RT-PCR (qPCR)

Reverse transcription was performed from total RNA using the High Capacity cDNA Reverse Transcription kit (Applied Biosystems, Carlsbad, CA), and transcript levels determined by means of Taqman Low Density Arrays (Applied Biosystems) on an ABI Prism 7900HT instrument (Applied Biosystems). Epithelial tumor cells were purified in selected cases by laser-microdissection (P.A.L.M. Mikrolaser, Bernried, Germany). A minimum of 2 mm^2^ of epithelium was obtained per sample, from which total mRNA was extracted using the RNeasy Micro Kit (Qiagen). Relative transcript quantification was determined by the ΔΔCt method using RPN18 transcripts as a reference and data were analyzed using the SDS 2.3 software (Applied Biosystems). ΔCt data were used to build hierarchical clusters by UPGMA (Unweighted Pair Group Method with Arithmetic Mean) [25]. The TaqMan assays used are detailed in Table S2 of File S1.

### Immunohistochemical analysis

Samples were deparaffinized and rehydrated prior to antigen retrieval, followed by incubation with primary antibodies (Table S3 of File S1), incubation with polymer-peroxidase-conjugated secondary antibody and developed with diaminobenzidine. Slides were counterstained with haematoxylin, dehydrated and coverslipped. This process was performed in an automatic immunostainer (Bond Automated Immunohistochemistry & In-Situ Hybridisation System). The reaction specificity was ascertained by the absence of staining when using a non-specific isotype-matched primary antibody.

 Nuclear immunostaining for ERα, PR and GATA3 was evaluated according to intensity (I; 0, 1, 2 and 3) and percentages of positive cells with the following discrete intervals (P): 0, 1 (1 - 9%), 2 (10 - 49%), 3 (50 - 74%) and 4 (75 - 100%). The final staining histoscore (Hscore) was obtained by multiplying intensity and percentage interval values (Hscore = I x P), thus ranging from 0 to 12. Hscores ≥ 2 were considered as positive staining. Membrane staining for HER2 was assessed following recommendations of the American College of Pathologists [26] as 0, 1, 2 and 3. Only cases with HER2 score equal to 3 were considered HER2(+). Primary tumors were classified according to the expression of hormone receptors and HER2. Cases were considered “luminal” with positive ER and/or PR (≥ 10% positive cells; ≥ 2 in our discrete scoring system) and negative HER2 (≤ 2); HER2(+) for cases with a immunohistochemical HER2 score of 3, irrespective of ER or PR; and “triple negative” (TN) when the three markers were negative.

### Statistical analysis

Data were analyzed using the SPSS package version 13.0 (SPSS Inc., Chicago, IL). Kruskal-Wallis test was applied in comparisons of means among groups, Mann-Whitney test for two-group comparisons and Chi square and Fisher’s exact test when analyzing categories. Differences were considered significant when *P* ≤ 0.05. Kaplan-Meyer curves and log-rank tests were used to assess metastasis-free survival (MFS), defined as the time elapsed from diagnosis to detection of distant metastases. 

## Results

### Expression profiling of node-positive vs. node-negative ductal breast cancers

Although lymph node metastasis is a major prognostic factor in breast cancer, little is known about the molecular mechanisms that confer breast cancer cells the capacity to metastasize to regional lymph nodes. To study this process, we compared the transcriptional profiles of 18 node-positive primary tumors, along with their lymph node metastatic samples, with 7 primary tumors with no detectable lymph node involvement at surgery. 

**Figure 1:**
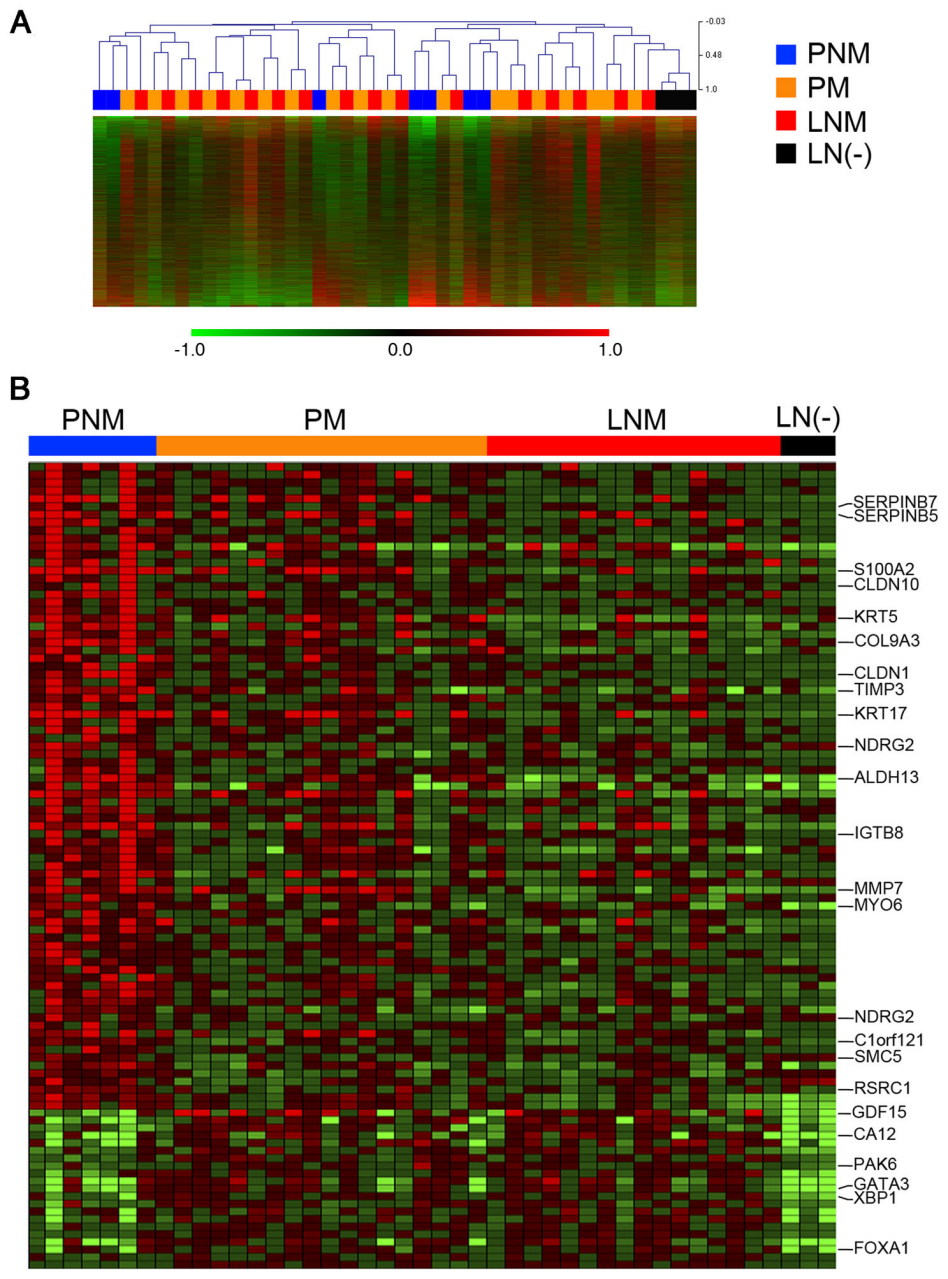
Identification by microarray analysis of genes differentially expressed between node-positive, node-negative and lymph node metastatic breast cancer samples. (A) Unsupervised analysis of microarray expression data for node-negative primary tumors (PNM), node-positive primary tumors (PM) and regional lymph node metastases matched to their primary tumors (LNM). (B) Supervised analysis showing genes differentially expressed between types of samples.

Unsupervised analysis of the microarray data failed to yield a clear-cut separation between the three classes of samples, namely primary node-negative (PNM), primary node-positive (PM) and lymph node metastases (LNM). In fact, node-positive primary tumors tended to pair with their matched lymph node metastases rather than form clusters with other node-positive primary tumors (Figure 1A), suggesting that there are no major differences in global gene expression patterns between node-positive and node-negative primary ductal breast cancer. A supervised approach was taken in an attempt to identify specific groups of transcripts whose levels distinguish these classes of samples. At a significance level of P ≤ 0.05 and fold changes in log_2_ of expression values ranging between 2.5 and -2.5, 107 genes were expressed at higher levels and 16 at lower levels in PNM vs. PM (Figure 1B). Many of the genes expressed at significantly higher levels in lymph node metastases than in primary tumors correspond to lymphoid and/or myeloid differentiation genes, suggesting that they merely reflect partial contamination of these samples with hematopoietic cells.

**Figure 2:**
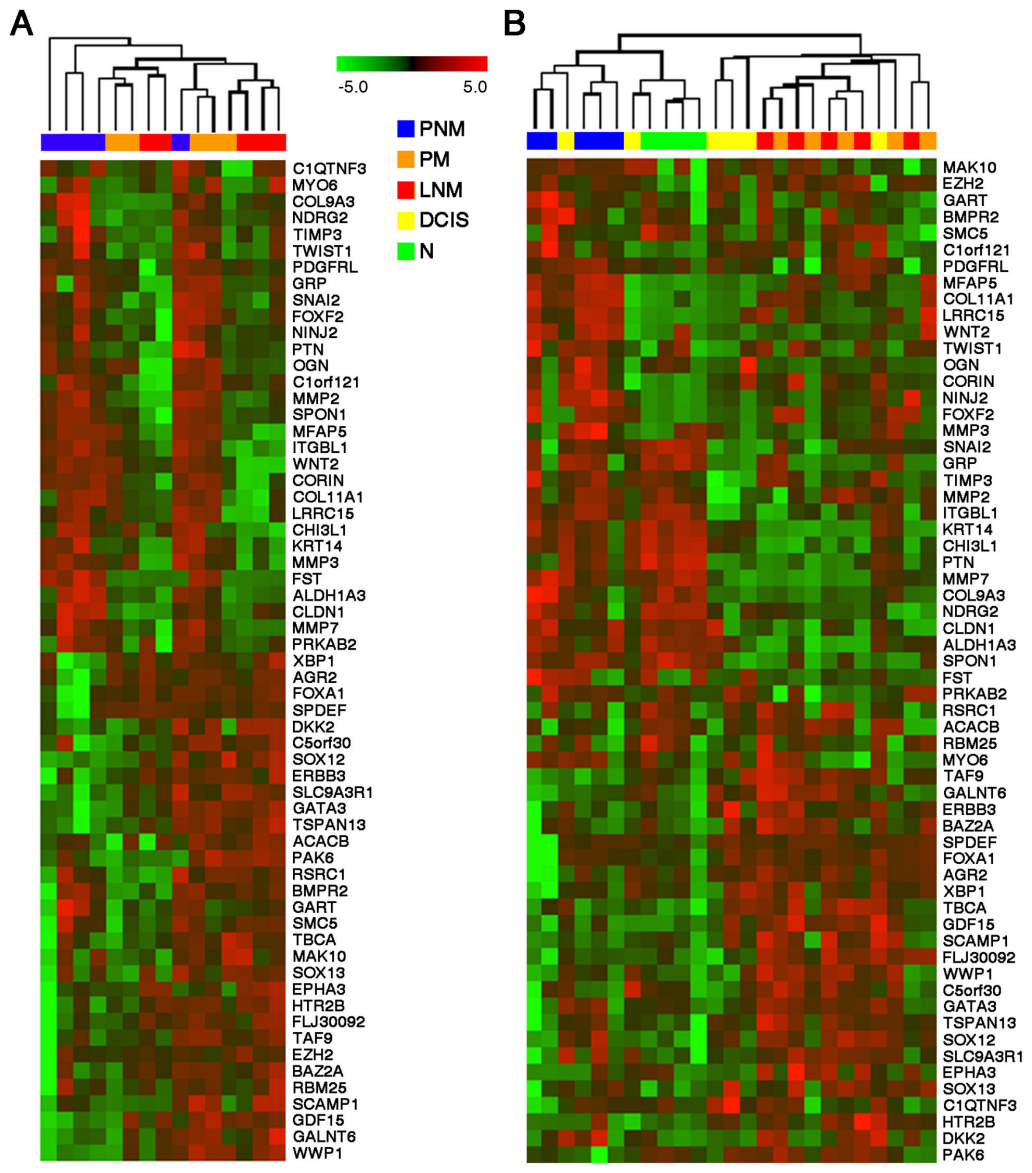
Expression levels of selected genes that discriminate between node-positive and node-negative breast cancer samples. (A) Hierarchical clustering based on QRT-PCR values for 61 genes in whole tissue samples. All but one PNM tumors were correctly segregated from PM tumors. (B) Hierarchical clustering based on QRT-PCR values for 61 genes in microdissected samples. Ductal carcinoma in situ (DCIS) samples were analyzed in addition to the sample categories shown in (A). Both analyses show that, among other genes, GATA3 is intensely expressed both in primary tumors and LNM samples.

Of the genes differentially expressed between node-positive and node-negative primary tumors, 61 were further analyzed by qPCR in 5 PNM and 5 PM tumors as well as 5 LNM, in order to confirm their differential expression. These genes were selected according to their capacity to discriminate between groups of samples (node-positive vs. node-negative primary tumors and primary vs. metastatic samples). Additional genes were also analyzed based on their established or proposed roles in breast cancer metastatic progression. All but one PNM samples were segregated from PM indicating that these genes can distinguish node-negative from node-positive primary tumors (Figure 2A). Additionally, the expression levels for the same 61 genes were also quantified in laser-microdissected epithelial-specific samples from 4 non-tumoral (normal morphology epithelium), 6 DCIS, 5 PNM tumors, 5 PM tumors and 5 LNM samples. As with total non-microdissected tissues, the quantification of the 61 transcripts in microdissected samples was capable of relatively good discrimination between node-negative and node-positive samples (Figure 2B). Normal samples and two DCIS samples clustered together with node-negative primary tumors, whereas lymph node metastases clustered together with primary node-positive tumors and the rest of the DCIS samples. The latter result corroborated that node-positive primary tumors and their matched lymph node metastases display highly overlapping transcriptional repertoires.

### Infrequent loss of GATA3 and ER expression in node-positive ductal breast cancers and metastases

Our comparative transcriptomic analysis yielded relatively few differentially expressed transcripts between node-positive and node-negative ductal breast tumors, which precluded significant inferences of associated signaling pathways or gene networks. Nevertheless, we noted that the luminal differentiation factor GATA3 was expressed at relatively high levels both in PM and in PNM samples, as well as in matched lymph node metastases (Figures 1 and 2). The levels of other transcripts associated with breast luminal epithelial differentiation, including ER, FOXA1 and XBP [27-31], followed a similar pattern, frequently with higher levels in node-positive than in node-negative primary tumors, and maintenance of levels in lymph node metastases (Figure 2). Analysis of transcript co-regulation in our samples showed that additional genes are expressed in parallel with GATA3, including those coding for carbonic anhydrase XII (CA12), cyclin D1 (CCND1) or hepsin (HPN) (Table S4 of File S1), all of which have been associated with breast cancer [32-35].

 The observed increase or maintenance in the expression levels of GATA3 along with metastatic progression in ductal breast cancer was unexpected, because this transcription factor is widely considered a metastasis and tumor suppressor by virtue of its function as a promoter of the commitment of progenitor breast epithelial cells to a differentiated luminal phenotype [36-40]. We thus extended our analysis to examine the expression of GATA3 and ER by immunohistochemistry in a larger set of formalin-fixed paraffin-embedded tissue samples. The examined tumors were stratified according to their ER, PR and HER2 status as luminal (ER and/or PR positive, HER2 negative), HER2 (HER2 positive) or triple negative (ER, PR and HER2 negative) (Table S1). 

**Figure 3:**
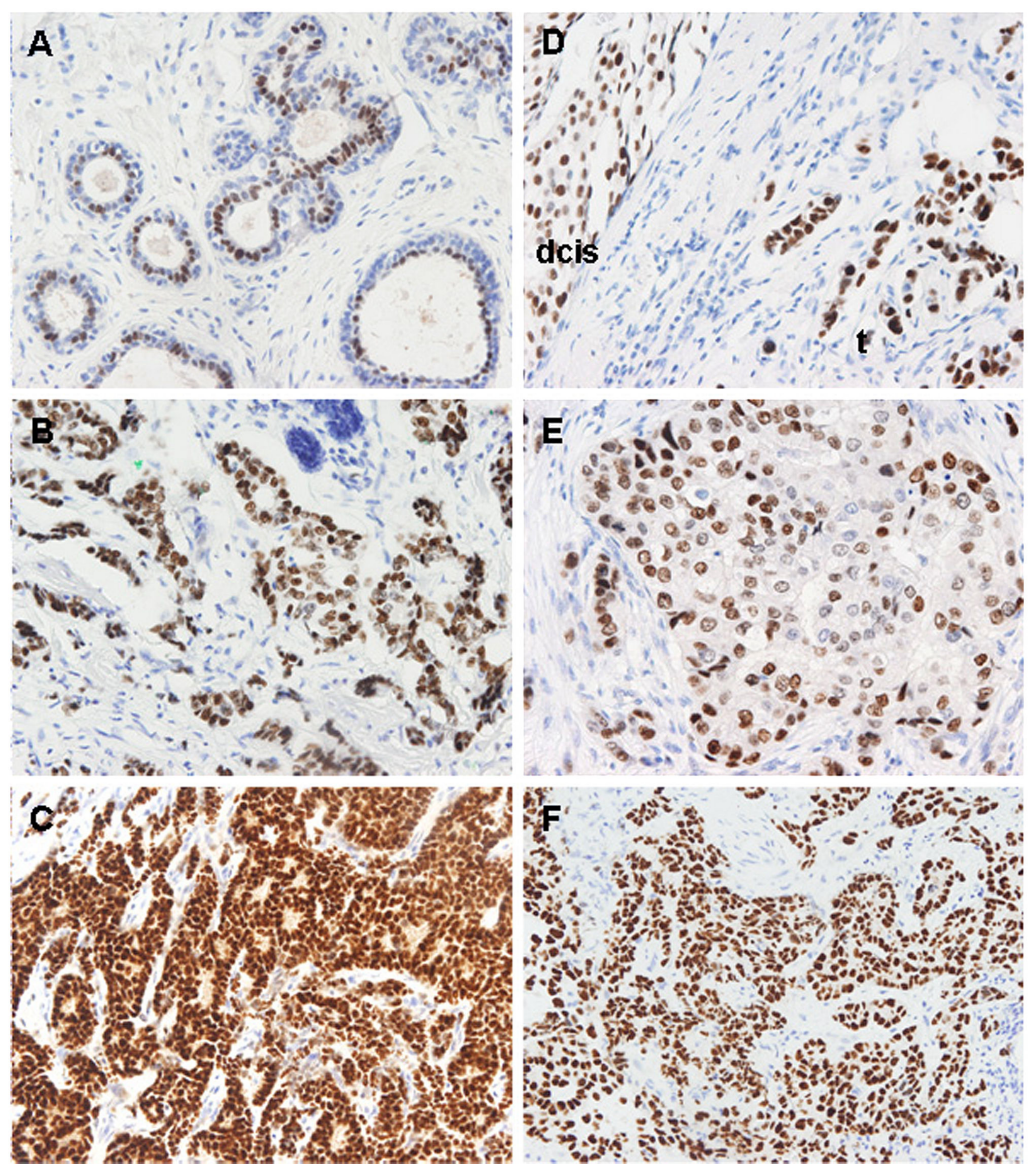
GATA3 immunohistochemical staining patterns in different types of breast samples and metastases. (A) The epithelium of ducts with normal morphology shows predominant GATA3 nuclear staining in the luminal layer. (B and C). GATA3 staining in a primary tumor and its metastasis to lymph node from the same patient than A. Tumor cells are strongly positive for GATA3 both in the primary (B) and its matched lymph node metastasis (C). (D) Stronger GATA3 staining in infiltrating carcinoma (t) as compared to its associated ductal carcinoma in situ (dcis). (E) Weak GATA3 staining of a node-negative breast cancer. (F) Intense GATA3 expression in a lung metastasis of a luminal breast cancer. Most tumor cells show intense positivity.

**Figure 4:**
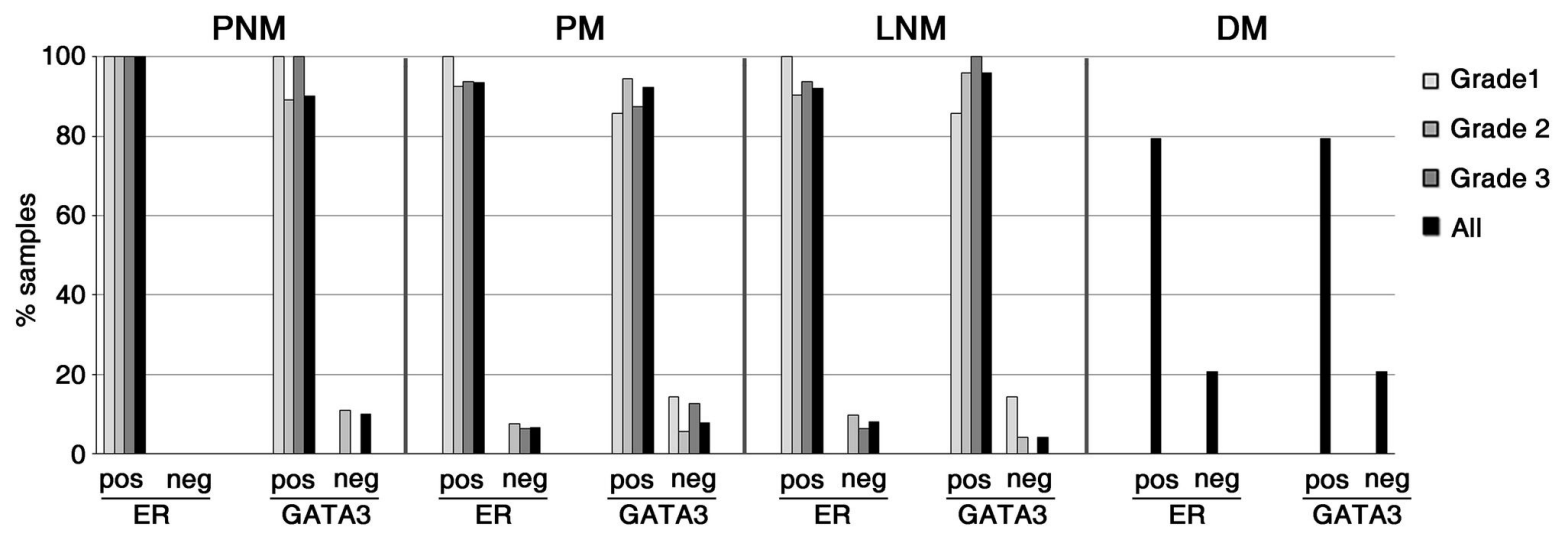
Maintenance of GATA3 and ER expression in lymph node metastases of luminal breast cancers. Samples from node-negative (PNM) or node-positive (PM) primary tumors, regional lymph node metastases (LNM) and distant metastases (DM) were immunostained for ER and GATA3, and assigned positive or negative status on the basis of Hscores.

We observed a predominantly nuclear staining for GATA3 in luminal cells of normal breast tissue (Figure 3A) [41], generally with a stronger intensity in malignant tissues than in normal tissues in matched (Figure 3A-C) and unmatched samples (Figure 3A, D-F) (Table S1). In cases with a luminal phenotype, we observed that the frequency of ER or GATA3 positive samples showed little variation between PMN, PM or LNM, independent of the histological grade of the primary tumor (Figure 4). About 80% of HER2(-) distant metastases expressed ER, GATA3 or both (Figure 4). In HER2(+) cases, the proportion of ER(-) samples was higher than in luminal cases, reaching to nearly 50% of PM and their matched LNM samples (Figure S1 of File S1). However, the proportion of GATA3(-) samples was similar to that of luminal cases, about 15% in HER2-positive PM and their matched LNM samples (Figure S1 of File S1). This suggests an uncoupling of the expression of these two luminal markers in these cases. In triple-negative cases, all PNM tumors were also GATA3(-) (Table S1), as expected from their presumed basal-like phenotype [42]. However, 4 of the 12 triple-negative PM tumors were GATA3(+), and this positivity was maintained in their matched lymph node metastases, again suggesting that expression of GATA3 can occur independently of ER and PR, in agreement with other reports [43].

**Figure 5:**
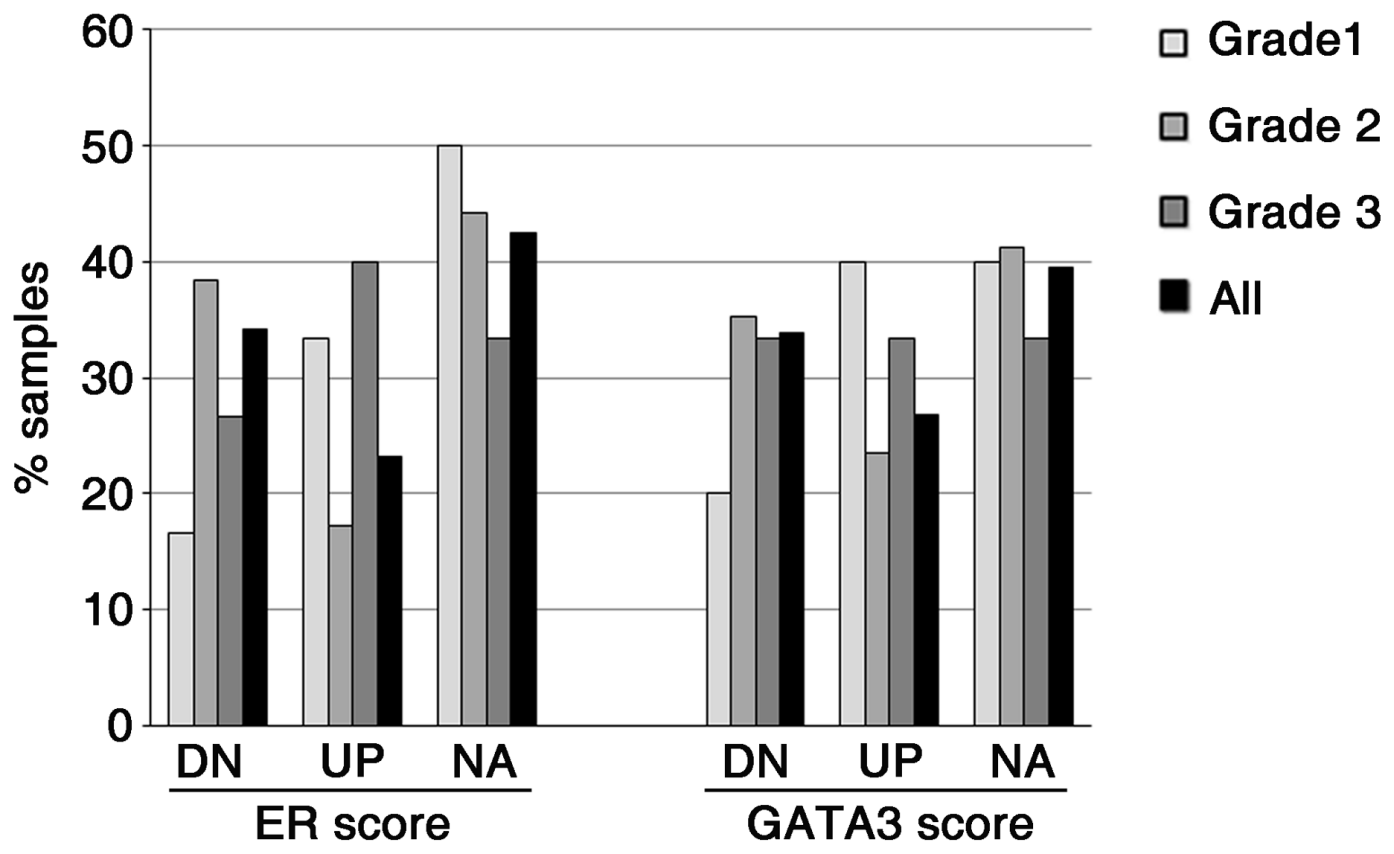
Variations in GATA3 and ER expression in node-positive luminal breast tumors and matched lymph node metastases. A majority of node-positive luminal-phenotype primary breast cancer cases maintains or increases the expression levels of ER and GATA3 in their matched lymph node metastases, independent of histological grade. A 20% decrease in Hscores from PM to its matched LNM was considered a downregulation (DN), a 20% increase an upregulation (UP) and Hscore variations inferior to 20% were considered as not altered (NA).

These results suggest that loss of luminal differentiation, as determined by loss or decline of ER and/or GATA3 expression, is infrequent in luminal-phenotype ductal breast cancer, irrespective of regional lymph node status and histological grade. To better assess the expression levels of ER and GATA3 along the metastatic progression of these tumors, we determined the Hscores for these markers in matched node-positive primary tumors (PM) and their corresponding lymph node metastases (LNM). In tumors with a luminal phenotype, we observed a decline in ER or GATA3 Hscores in about 30% of the LNM samples when compared to their matched PM (Figure 5). The remaining 70% of the samples had equal or higher Hscores in LNM samples compared to their matched PM samples. Of further interest, although the expression of GATA3 and ER was generally concordant between primary tumors and their matched lymph node metastases, 3 of the luminal-phenotype PM cases switched from GATA3(-) in PM to GATA3(+) in their matched lymph node samples, and 3 of the GATA(+) luminal PM cases became GATA3(-) in their matched LNM. Similarly, 3 cases in this group switched from ER(-) in PM to ER(+) in their matched LNM, and 3 other ER(+) cases switched to ER(-) in their matched LNM (Table S1). These switches in GATA3 expression between PM and LNM were not necessarily concordant with the switches in ER expression. This further highlights that the expression of GATA3 can uncouple from ER in metastatic transitions.

**Figure 6:**
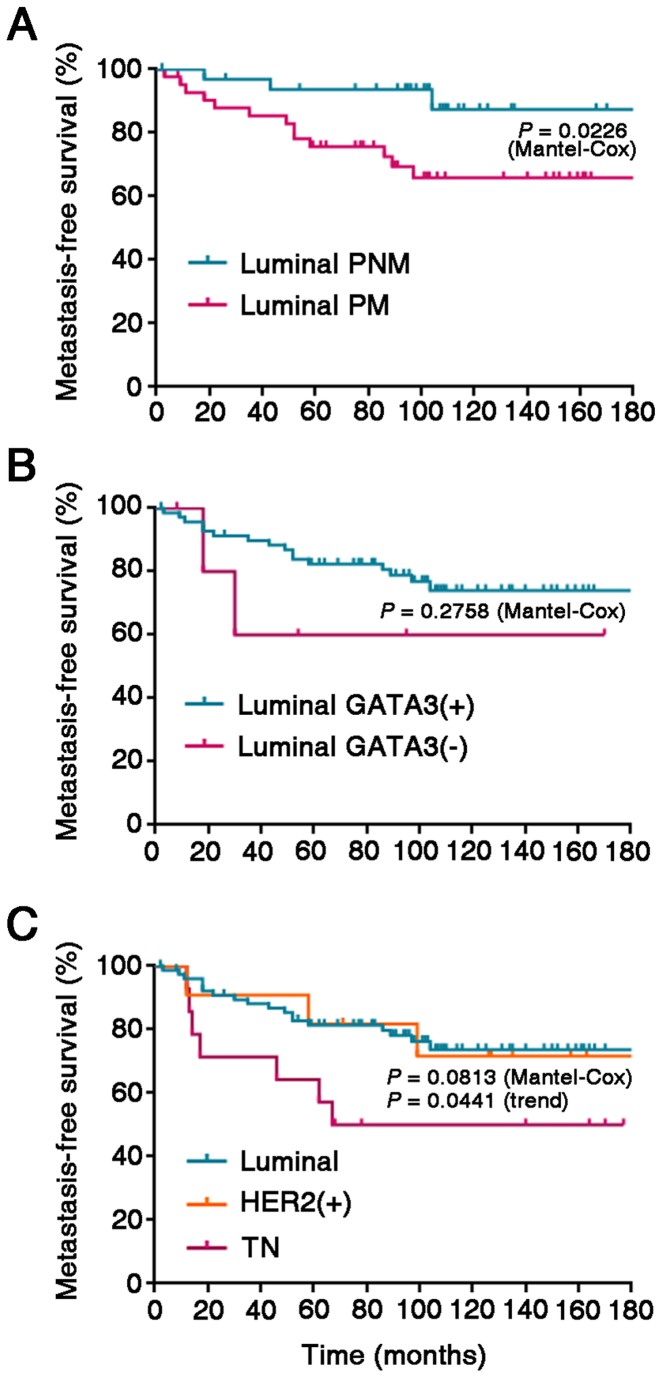
Metastasis-free survival curves for luminal phenotype cases as a function of GATA3 expression status. (A) Kaplan-Meier metastasis-free survival actuarial curves for PNM vs. PM cases. (B) Metastasis-free survival actuarial curves according to GATA3 status. (C) Metastasis-free survival actuarial curves according to molecular phenotype (luminal, HER2(+), TN).

 Our observations corroborate that metastasis to local and regional lymph nodes of luminal-type breast cancer only infrequently involves a decline in the expression of ER or GATA3. As expected, actuarial survival curves showed that node-positive luminal cases (PM) developed distant metastases at significantly earlier times than node-negative cases (Figure 6a). Although relatively few cases with a luminal primary tumor phenotype were GATA3(-), which precluded reaching statistical significance, these cases also tended to present worse distant metastasis-free survival than GATA3(+) cases (Figure 6b), in agreement with previous work [38,44-46]. Also as expected, triple-negative cases had a worse distant metastasis-free survival than luminal tumors (Figure 6c).

## Discussion

Although primary breast tumors are often successfully treated, the emergence of metastases is often life-threatening. As in other carcinomas, the dissemination of cells from the primary tumor is first detected in regional lymph nodes, the involvement of axillary lymph nodes being a major prognostic factor in breast cancer [15,17,19]. In order to acquire a better insight into the process of lymph node metastasis in ductal breast cancer, we conducted a microarray-based transcriptomic survey, followed by real-time PCR quantification of selected transcripts and immunohistochemical analysis on a larger panel of samples. Our analysis leads us to propose that luminal differentiation, as assessed by estrogen receptor and GATA3 expression levels, is only infrequently lost during the process of regional lymph node colonization by breast cancer cells. Alternatively, transient downregulation of the luminal gene program might take place at the primary tumor site in association with local invasion and intravasation, followed by re-expression of the differentiation program after tumor cells colonize lymph nodes or distant organs. In either case, our results suggest that, in general, metastatic luminal-phenotype tumors are not characterized by a stable loss of their differentiated features. More specifically, loss of GATA3 expression does not appear to be a requirement for the establishment of metastatic growth in a majority of luminal breast cancers.

Even though it is not recognized as a “canonical” hallmark of cancer [47], loss of differentiation constitutes a distinctive feature of many tumors and an indicator of malignant progression [48] and the basis for therapies aimed at restoring differentiated properties of tumor cells [49,50]. The discovery that loss of GATA3, a critical factor for luminal lineage determination during breast epithelial differentiation [36,39], can drive tumorigenesis in experimental models [37,38] and correlates with poor prognosis in breast cancer patients [40,45,46,51] justifiably prompted its consideration as a tumor suppressor, as well as expectations that new therapeutic strategies could be devised to induce differentiated features in breast cancer cells [52]. 

In agreement with its function as a determinant of breast epithelium luminal differentiation, we observed low levels of GATA3 levels in triple-negative breast cancer cases, a majority of which are likely to correspond to basal-like phenotypes [42]. However, we also found that the expression levels of GATA3 and ER were only infrequently diminished in node-positive primary ductal cancer as compared to node-negative tumors. Moreover, only about 20% of cases showed a decline in the expression of these luminal markers in lymph node metastases as compared to their matched primary tumors, independent of histological grade. In distant metastases, over 70% of HER2(-) samples expressed ER, GATA3 or both. The latter observations are in agreement with a recently published report [43] showing that the majority of breast cancer distant metastases, including those with luminal and triple-negative phenotypes in their primary tumors, maintain GATA3 expression. Therefore, our observations suggest that loss of luminal differentiation may not play a significant role as a driver of lymph node or distant metastasis in a majority of cases of luminal breast cancer. In spite of the observed infrequent loss of luminal differentiation markers in distant metastases, loss of GATA3 expression in primary tumors with an otherwise luminal phenotype (positive for ER and/or PR and negative for HER2) was correlated with a poor metastasis-free survival of patients, in agreement with previous studies [40,45,46,51].

These observations also raise the question whether GATA3 is a true metastasis suppressor in luminal breast cancer [37,51,53,54]. Using mouse models, Kouros-Mehr et al. [38] showed that overexpression of GATA3 in cells from incipient tumors (adenomas) inhibited their capacity to disseminate after transplantation, although no evidence was provided as to whether the establishment of metastasis was enhanced, an attribute of tumor cells distinct from their capacity to disseminate. In the same report, conditional knock out of GATA3 in more advanced tumor cells led to their apoptosis, indicating that GATA3 was required for the survival of advanced-stage tumor cells. Moreover, those tumors that eventually developed reexpressed GATA3. The authors suggested that such tumors were formed from cells that had escaped GATA3 knockout [38], suggesting again that those tumor cells require GATA3 in order to survive.

A putative growth-promoting or maintenance function of GATA3 might be performed through its transcriptional repression of the cyclin-dependent kinase inhibitor p18^INK4c^ [55]. In addition, GATA3 appears to promote carcinogenesis in a lymphoma model induced by the increased expression of c-Myc [56,57], which in turn induces Notch1 and GATA3 as putative transcriptional targets that cooperate with Myc to establish a malignant phenotype [58]. Likewise, GATA3 can be overexpressed in pancreatic cell lines and primary pancreatic cancers [41], and in neuroblastoma cell lines [59], where it positively regulates cyclin D1, maintaining cells in an undifferentiated state [59], therefore suggesting an oncogenic potential for GATA3 in neuroblastoma. A further mechanism by which GATA3 expression might sustain cell growth is through the positive regulation of the proto-oncogenic aurora-kinase A in response to estrogen in ER-positive breast cancer cells [60].

Therefore, experimental and correlative evidences suggest a positive role for GATA3 in the survival and maintenance of the proliferative state of normal and neoplastic breast epithelial cells. This, together with our observations and those of Cimino-Mathews et al. [43] that breast cancer metastases generally maintain the expression of GATA3, leads us to suggest that current views of GATA3 as a putative tumor and/or metastasis suppressor in these tumors merit re-examination.

## Supporting Information

Table S1 **Immunohistochemical results for luminal markers and Her2.**
(XLSX)

File S1 **Additional tables S2, S3 and S4.** Additional Figure S1.
(DOC)
