# Supplementary material for: Infrequent Loss of Luminal Differentiation in Ductal Breast Cancer Metastasis
Source: PLoS One. 2013 Oct 21;8(10):e78097. doi: 10.1371/journal.pone.0078097 (PMC3804564; doi:10.1371/journal.pone.0078097)
Supplement: File S1 — Additional tables S2, S3 and S4. Additional Figure S1. (DOC) [file pone.0078097.s002.doc]

# Supplementary information

# Infrequent loss of luminal differentiation in ductal breast cancer metastasis

Julia Calvo^1^, Lourdes Sánchez-Cid^1,2^, Montserrat Muñoz^3,4^, Juan José Lozano^5,6^, Timothy M. Thomson^2,7^* and Pedro L Fernández^1,4,8^*.

^1^  Department of Pathology, Hospital Clínic, Barcelona, Spain.

^2^ Department of Cell Biology, Molecular Biology Institute of Barcelona (IBMB), National Research Council (CSIC), Barcelona, Spain.

^3^ Department of Medical Oncology, Hospital Clínic, Barcelona, Spain

^4^ University of Barcelona, Barcelona, Spain

^5^ Plataforma de Bioinformática, Centro de Investigación Biomédica en Red de Enfermedades Hepáticas y Digestivas (CIBER-EHD), Hospital Clinic, Barcelona, Spain

^6^ Centre d’ Investigacions Esther Koplowitz (CEK), Barcelona,Spain

^7^ Networking Research Centre for Bioengineering, Biomaterials and Nanomedicine (CIBER-BBN), Instituto de Salud Carlos III, Zaragoza, Spain

^8^ Institut d’Investigacions Biomèdiques August Pi i Sunyer, Barcelona, Spain

Contents:

Supplementary Tables S2-4 and Supplementary Figure S1 with Legend

**Supplementary Table S2**. TaqMan assays used for qPCR.

| **Gene Symbol** | **Assay ID** | **Amplicon size** |
| --- | --- | --- |
| 18S (control) | Hs99999901_s1 | 187 |
| ACACB | Hs00153715_m1 | 88 |
| AGR2 | Hs00180702_m1 | 107 |
| ALDH1A3 | Hs00167476_m1 | 60 |
| BAZ2A | Hs00203782_m1 | 108 |
| BMPR2 | Hs00176148_m1 | 69 |
| C1orf121 (FAM152A) | Hs00274995_m1 | 108 |
| C1QTNF3 | Hs00229569_m1 | 65 |
| C5orf30 | Hs00264241_m1 | 119 |
| CHI3L1 | Hs00609691_m1 | 77 |
| CLDN1 | Hs00221623_m1 | 82 |
| COL11A1 | Hs00266273_m1 | 98 |
| COL9A3 | Hs00156761_m1 | 110 |
| CORIN | Hs00198141_m1 | 102 |
| DKK2 | Hs00205294_m1 | 88 |
| EPHA3 | Hs00178327_m1 | 66 |
| ERBB3 | Hs00176538_m1 | 62 |
| EZH2 | Hs00544830_m1 | 86 |
| FLJ30092 (C12orf51) | Hs00383293_m1 | 66 |
| FOXA1 | Hs00270129_m1 | 74 |
| FOXF2 | Hs00230963_m1 | 77 |
| FST | Hs00246260_m1 | 105 |
| GALNT6 | Hs00200529_m1 | 85 |
| GART | Hs00531926_m1 | 81 |
| GATA3 | Hs00231122_m1 | 80 |
| GDF15 | Hs00171132_m1 | 78 |
| GRP | Hs00181852_m1 | 86 |
| HRH1 | Hs00185542_m1 | 96 |
| HTR2B | Hs00168362_m1 | 70 |
| ITGBL1 | Hs00191224_m1 | 66 |
| KRT14 | Hs00265033_m1 | 64 |
| LRRC15 | Hs00370056_s1 | 89 |
| MAK10 | Hs00257238_m1 | 86 |
| MFAP5 | Hs00185803_m1 | 82 |
| MMP2 | Hs00234422_m1 | 83 |
| MMP3 | Hs00968308_m1 | 98 |
| MMP7 | Hs00159163_m1 | 101 |
| MYO6 | Hs00192265_m1 | 110 |
| NDRG2 | Hs00212263_m1 | 94 |
| NINJ2 | Hs00356576_m1 | 89 |
| OGN | Hs00247901_m1 | 71 |
| PAK6 | Hs00220131_m1 | 66 |
| PDGFRL | Hs00185122_m1 | 69 |
| PITX2 | Hs00165626_m1 | 79 |
| PRKAB2 | Hs00271294_m1 | 70 |
| PTN | Hs00383235_m1 | 76 |
| RBM25 | Hs00402198_m1 | 74 |
| RSRC1 | Hs00275476_m1 | 78 |
| SCAMP1 | Hs00191607_m1 | 65 |
| SLC9A3R1 | Hs00188594_m1 | 71 |
| SMC5 | Hs00390892_m1 | 69 |
| SNAI2 | Hs00161904_m1 | 79 |
| SOX12 | Hs00272869_s1 | 80 |
| SOX13 | Hs00232193_m1 | 70 |
| SPDEF | Hs00171942_m1 | 68 |
| SPON1 | Hs00323883_m1 | 76 |
| TAF9 | Hs00852909_g1 | 149 |
| TBCA | Hs00855137_g1 | 148 |
| TIMP3 | Hs00165949_m1 | 59 |
| TSPAN13 | Hs00205231_m1 | 63 |
| TWIST1 | Hs00361186_m1 | 115 |
| WNT2 | Hs00608224_m1 | 119 |
| WWP1 | Hs00366927_m1 | 70 |
| XBP1 | Hs00231936_m1 | 60 |

**SupplementaryTable S3**. Source, type of antigen retrieval, dilution and incubation time used for each primary antibody

| **Antigen** | **Clone** | **Source** | **Retrieval pH/time at max. temp** | **Dilution** | **Incubation time/temp.** |
| --- | --- | --- | --- | --- | --- |
| **ERα** | 1D5 | DAKO | pH6/ 20' | 1/100 | 60 min/ RT |
| **PR** | 16 | Novocastra | pH6/ 20' | 1/100 | 60 min/ RT |
| **GATA3** | HG3-31 | Santa Cruz Biotechnology | pH9/ 15' | 1/200 | 120min/ RT |
| **c-erbB2** | PolyclonalA0485 | DAKO | pH9/ 15' | 1/700 | 60 min/ RT |
|  |  |  |  |  |  |

RT: room temperature

**Supplementary Table S4**. Top 50 genes co-regulated with GATA3 showing *r* values for 3 different GATA3 Affymetrix probes.

**Supplementary Figure S1**

**Supplementary Figure S1**. Primary and metastatic samples positive for HER2 immunostaining show more frequent ER negative status as compared to samples with a luminal phenotype (main Figure 4), but tend to maintain GATA3 expression in primary tumors (PM), their matched lymph node metastases (LNM) and in distant metastases (DM).
